# The impact of the COVID-19 lockdown on depression sufferers: a qualitative study from the province of Zaragoza, Spain

**DOI:** 10.1186/s12889-022-13083-2

**Published:** 2022-04-18

**Authors:** Alejandra Aguilar-Latorre, Bárbara Oliván-Blázquez, Ana Porroche-Escudero, Fátima Méndez-López, Valentín García-Gallego, Belén Benedé-Azagra, Rosa Magallón-Botaya

**Affiliations:** 1 Institute for Health Research Aragón (IIS Aragón), Zaragoza, Spain; 2 Department of Psychology and Sociology, University of Zaragoza, Zaragoza, Spain; 3 Lancaster Environment Centre, Lancaster University, Lancaster, UK; 4 Aragonese Healthcare Service (SALUD), Zaragoza, Spain; 5 Department of Medicine, Psychiatry and Dermatology, University of Zaragoza, Zaragoza, Spain

**Keywords:** Social determinants of health, Depression, COVID-19, Qualitative study

## Abstract

**Background and purpose:**

The impact of COVID-19 and its control measures have exacerbated existing mental health conditions. Although the deleterious effects of mental health problems are well known, fewer studies have examined the links between the Social Determinants of Health (SDHs) and depression. This study provides insights into the relationship between SDHs and depression during the first strict lockdown in Spain, which lasted for a period of 7 weeks.

**Methods:**

Fifty-two structured interviews were conducted with people diagnosed with depression during June 2020 in the province of Zaragoza (Spain). Interviews were conducted by telephone due to lockdown constraints. Inductive thematic content analysis was used to explore, develop, and define emergent categories of analysis, which were mapped against the SDH framework.

**Results:**

Listening to people’s experiences of living with depression during lockdown provided insights into their concerns and coping strategies, which are greatly influenced by the conditions in which they live, their job and their age. Examples of these factors include access to and quality of physical spaces, including housing conditions and public spaces for socialising, social support, adverse working conditions which include caring responsibilities, and access to digital technologies and healthcare services.

**Conclusion:**

SDHs have played a fundamental role in shaping people’s health and well-being during the COVID-19 pandemic, and this study has shown that they have a considerable effect on depression outcomes. Governments should consider implementing social welfare programs to tackle both psychosocial problems and material need during crisis situations.

## Introduction

Depression is considered to be the main worldwide cause of disability, contributing to the overall global burden of morbidity and mortality. By 2030, it is expected to be the main contributor to the burden of morbidity [1–3]. The onset and continuation of depression has been linked to numerous biological and psychosocial factors, many of which are related to different aspects of lifestyle [4]. Individual lifestyles are embedded in social and community networks and living and working conditions, related to the broader cultural and socio-economic environment [5]. Socio-economic status, education, neighbourhood and physical environment, employment, and social support networks, as well as access to healthcare, are all Social Determinants of Health (SDHs) [6, 7]. SDHs have proven crucial for our mental health [5, 8]. Addressing SDHs is essential to improving health and reducing inequalities in health and healthcare [9]. Social analysis of health problems was evidenced in the Lalonde Report of Canada in 1974 [10]. This report proposed that we understand health as a fundamental human right, accepting the following as fundamental conditions for health: peace, education, housing, food, income, a stable ecosystem, social justice and equity [11, 12].

The coronavirus disease 2019 (COVID-19) caused by Severe Acute Respiratory Syndrome SARS-CoV-2 has caused a devastating and unprecedented worldwide health, social and economic crisis [13, 14]. Governments around the world have implemented wide-ranging public health emergency measures to cope with rapidly spreading outbreaks. These measures have included self-isolation, curfews and stay-at-home orders, physical distancing, travel restrictions, as well as the closing of borders, schools, stores, restaurants, and workplaces, as well as the cancellation of public events [15, 16].

The impact of COVID-19 and its control measures on mental well-being cannot be underestimated. Amounting evidence suggests that the pandemic has exacerbated existing mental health conditions and triggered new ones [17–20]. Physical distancing measures and social isolation have been linked to common psychological symptoms such as boredom, stress, anxiety, depression, disrupted sleep and feelings of helplessness [21–23]. It is well established that social support provides important social, emotional, and material resources, and in doing so offers a buffer against distress [24]. Thus, long periods of uncertainty and insecurity, compounded by loneliness and isolation, have been significantly associated with mental disorders and poor mental well-being [25].

Socially vulnerable groups hit harder by COVID-19 include those with pre-existing mental disorders and chronic physical diseases, frontline workers, infected or suspected patients, those living in areas with high incidence, and those who are financially less well-off [26]. It is increasingly demonstrable that SDHs such as working and living conditions, including the physical environment (e.g., housing), have had a considerable impact on mental health. SDHs have interacted with gender [27], ethnicity, class and other factors to increase COVID-19 related inequalities [28, 29]. For instance, people living in socio-economically disadvantaged neighbourhoods tend to work in low-income jobs which often entail physical proximity to other people, direct contact with the public or a lack of power to demand safe protective equipment or sick leave, thus increasing exposure to risk [30]. These people are also more likely to live in poorer quality housing (e.g. poor ventilation; poor control over housing conditions in rented flats) with higher density occupancy, which also increases the risk of transmission [30–32].

Furthermore, low income may impact on people’s ability to access the internet and communication technologies such as tablets, smartphones, and laptops, as some families cannot afford such devices. This situation can exacerbate inequalities by preventing people from accessing information about the pandemic, impacting on diagnosis and follow-up [32]. Digital exclusion also reduces “social connectivity”, which is supposed to promote opportunities [33]. For instance, lack of digital access will prevent people from learning about employment opportunities and their employment rights. Insufficient social services and other public resources for socio-economic support are more likely to affect low-income earners (i.e., people continue in physical work for fear of losing their source of income). In particular, people living with mental health problems and other conditions are disproportionately affected by socio-economic inequalities, impacting on their ability to comply with quarantine rules. Some people find it impossible to isolate due to the lack of a care network for people with mental illness, as do those without a family or who have been affected by the closure of day centres or suspension of social activities [32].

COVID-19 related morbidity and mortality have been unevenly distributed across geographic areas. For instance, Spain is one of the countries most affected by the disease [32], and it implemented one of the strictest lockdowns in Europe for seven weeks. During the first “State of Alarm” imposed by the central government (provisionally from March 15 to 29), people’s movements were restricted to trips to specific outlets to, for example, acquire food and medicine, attend healthcare appointments, go to work, and due to force majeure (internal borders were closed a week later). During the first imposition of the stay-at-home lockdown (from March 30 to April 12), Spain rose to third and second place in the world in terms of confirmed diagnoses and catastrophic losses, respectively. After six extensions of lockdown (the last on June 7) and four transition stages in which they contemplated somewhat more relaxed measures similar to other EU countries, Spain finally moved into the “new normal” on June 21, 2020 [34]. It is in the month of June 2020 when this study was carried out, after seven weeks of strict lockdown.

Mirroring a trend in health research [35], a large amount of COVID-19 studies tend to have a biomedical or epidemiological focus [36, 37], framing health problems in terms of behaviours and individual lifestyle risk factors. This focus neglects the role of SDHs in differential exposure to the virus, differential vulnerability to the infection and differential consequences of the disease. Qualitative methods can provide valuable insights into the experiences of people bearing the burden of COVID and the professionals managing the problem, as well as on the broader social and environmental conditions that facilitate the differential impact of COVID [38, 39]. Gaining this knowledge is essential to developing more effective prevention and management strategies. Yet, these studies are few and far between [17]. There are even fewer qualitative studies exploring the links between mental health, depression and COVID [40, 41]. This qualitative study provides insights into the relationship between SDHs and depression during the first strict lockdown in Zaragoza, Spain.

## Methods

### Study design

Fifty-two structured telephone interviews were conducted with people diagnosed with depression living in the province of Zaragoza, Spain. The SDH framework informed the interview guide. The questions were designed to explore people’s experiences holistically, without assuming that some SDHs were more important than others. Thus, the questions were general enough to allow participants to explore complex and interconnected factors (Table 1).

**Table 1:** Topic list

1. Personal and family COVID-19 situation: Have you or someone who lives with you been infected? Has someone close to you died? Who have you lived with during lockdown?
2. What type of home do you have? (Flat without terrace or balcony, flat with terrace/balcony, house with a garden, etc.)
3. How has your state of mind been during lockdown? What has caused you the most suffering? (i.e., not being able to go out, not being able to be with your loved ones, the images you saw of the pandemic, etc.)?
4. Do you think your state of mind is better or worse now compared to before lockdown? What do you think has made you feel better/worse? What has helped you to overcome this situation?
5. What do you think the health system could have done to help you with your mood during lockdown?

### Participant recruitment

Patients were recruited from an existing randomised controlled trial (RCT) on a lifestyle modification programme for patients with subclinical and major depression, led by the authors AAL, BOB and RMB [42]. A trained research assistant (RA) phoned potential participants to explain this qualitative study and the patient information sheet. If participants accepted and met the eligibility criteria the RA also obtained written informed consent and booked an appointment for the interview.

The eligibility criteria were that patients had to be 18 or over, and had to have been living with subclinical, mild, moderate, or severe depression for at least two months prior to lockdown. To determine participants’ mental health, the RA used the Beck II Self-Applied Depression Inventory (BDI II) [43]. Ability to understand written and spoken Spanish and provide written consent were also requirements.

The exclusion criteria were as follows: suffering from another disease that affects the brain (organic brain pathology or having suffered a traumatic brain injury of any severity, dementia); having another psychiatric diagnosis or severe psychiatric illness (substance dependence or abuse, a history of schizophrenia or other psychotic disorders, eating disorders), except for anxious pathology or personality disorders. This data was collected through a medical history and from the Mini-International Neuropsychiatric Interview (MINI) [44]. Other exclusion criteria were: the presence of a severe or uncontrolled medical, infectious or degenerative illness that may interfere with the affective symptoms; delirium or hallucinations, suicide risk, pregnancy or lactation; and the presence of any medical, psychological or social problem that could seriously interfere with the patient’s participation in the study.

### Data collection

AAL interviewed participants in June 2020. She introduced herself as a trained research psychologist and adhered strictly to the script to minimise her intervention in the interview process. Data collection was conducted by telephone due to lockdown restrictions. Interviews lasted no longer than 30 min. All sessions were digitally audio-recorded, and a verbatim transcription was made to obtain the final set of qualitative data for analysis. Participants agreed to participate in the study and signed a consent form.

### Data analysis

All personally identifiable information was removed from the transcripts and replaced with an anonymised personal unique identifier. We developed a system to enable record linkage between transcripts, socio-demographics and BDI II.

An inductive thematic content analysis was carried out to identify themes emerging from the interviews [45], while recognising that themes may also come from the preconceived SDH framework [46]. First, five researchers (AAL, FML, VG, RMB, APE) read through all the transcripts and identified emerging themes and potential subthemes which were agreed in team meetings. Secondly, four researchers (AAL, FML, VG, RMB) revised the scripts and mapped themes and subthemes against the SDH framework (Table 2). Participants’ quotations were extracted, and new themes were also identified. Thirdly, the team met to discuss discrepancies as well as new themes. The interpretations of the data were discussed with interviewers and participants to obtain their consent [47]. This methodological triangulation increased consistency and rigour by combining multiple techniques and maximising the interpretations’ breadth and depth. All analysis was performed iteratively using MAXQDA software (Qualitative Data Analysis) [48].

**Table 2:** Overview of the themes and subthemes mapped against the SDH framework

SDH	Themes and subthemes
Neighbourhood and physical environment	a. Public/private housing – access to the ‘outdoors’ within the home and quality of physical space—balcony b.The outdoors/street c.Outdoor exercise
Community, safety, and social context	d. Living together in the space e. Family and social support f. Extra work g. Digital technologies h. Home as a privilege and leisure-time activities
Healthcare services	

## Results

Participants’ demographic characteristics and COVID-19 infection information are presented in Table 3 in numbers and percentages. Most participants were adult women (88.5%), typically with secondary and primary education, married or living with a partner, not in employment and without short/long-term disability, receiving from 1 to 2 times the Interprofessional Minimum Wage (IMW) or less, living in a house with a small balcony or terrace. Most of the participants had not been infected with COVID-19 (96.15%). One had a family member that had been infected, and two had lost a relative to the virus.

**Table 3:** Characteristics of the participants

Variables	Patients (*n* = 52)
**Age**	53.45 (± 14.26)
Young adults (18 to 39 y/o)	10 (19.2%)
Middle-aged adults (40 to 49 y/o)	7 (13.5%)
Mature adults (> 50 y/o)	35 (67.3%)
**Gender**	
Male	6 (11.5%)
Female	46 (88.5%)
**Education**	
None	5 (9.6%)
Primary	21 (40.4%)
Secondary	23 (44.2%)
Tertiary	3 (5.8%)
**Occupation**	
Employed	8 (15.4%)
Unemployed	4 (7.7%)
Stay-at-home parent/unpaid worker/student/pensioner	20 (38.5%)
Sick leave/short/long-term disability/ other situations	20 (38.5%)
**Marital status**	
Single	5 (9,6%)
Married or living with a partner	31 (59.6%)
Separated, divorced or in separation proceedings	11 (21.1%)
Widower or widow	5 (9,6%)
**Income level**	
< IMW	23 (44.2%)
1 to 2 × IMW	25 (48.1%)
> 2 and < 4 × IMW	4 (7.7%)
**Type of home**	
Small apartment without a balcony or terrace	6 (11.54%)
Apartment with a small balcony	23 (44.23%)
Apartment with a terrace	18 (34.62%)
House with balconies or house with a large garden	5 (9.61%)
**COVID-19 Infections**	
Not infected	50 (96.15%)
Infected themselves	2 (3.85%)
Infected relative	1 (1.92%)
Loss of a relative due to COVID-19	2 (3.85%)

Note: *y/o* years old, *IMW* Interprofessional Minimum Wage

### Neighbourhood and physical environment

- Home balconies as an extension of the street

The physical space (availability and quality) of the home was an important factor shaping well-being. Most participants appreciated their terraces and balconies during lockdown. These spaces allowed them to “leave the house” and be outdoors, which became an extension of the street for many. The positive perception that “being on the balcony makes me feel that I am out on the street” was recurrent in those living in apartments or houses with balconies. Some participants used it to walk, others to socialise with the neighbours and others to distract themselves for a while (while reading, looking outside, etc.). At 8 pm, it was customary to go out to the balcony to applaud the health professionals. Several participants mentioned this time as something positive that served to unite the neighbours since they knew that at 8 pm, they were going to see each other, and they were going to spend some time together clapping and chatting. Participants expressed that a feeling of community union emerged in the face of adversity.

*Well, the radio provided me with company a lot of the time, and the ladies next door, or I’d just watch the balconies opposite, I used to watch life unfold on those balconies, I lived on my balcony, it was my comfort zone.* Woman, aged 56.

Only five patients live in a house with a balcony or a large garden. Two of them spent lockdown in their village, where people commonly live in a house with a large garden. They stated that having a piece of land made them feel like they were “in the countryside”. It is worth mentioning that houses with balconies, and in particular houses with gardens, are not common in urban Spain.

b. The role of the street and going out

The fact of being unable “to go out” (*salir*) or “leave the house” literally paralysed people’s routine. They could no longer go out to do their daily activities or meet their friends. Almost half of the participants reported that their situation worsened due to the change of routine and highlighted the importance of their daily routine:

*It has made me feel terrible. It has made everything worse for me. It seemed like it wasn’t going to affect me, but in the end it did. Not being able to go out, not being able to see the people you want to see… Nothing that would have helped could be done.* Woman, aged 42.

Since Zaragoza city has the highest rate of inward migration in the province, another recurrent complaint from people was not being able to go to their hometowns or villages (*pueblos*) to walk in the countryside and visit their families. Several participants acknowledged that they usually did not go out much, but the lack of choice made them want to go out more. For the youngest participants, online teaching and the cancelation of sport activities was emotionally challenging. In general, lockdown was perceived as the worst consequence of the pandemic for those used to doing outdoor activities. Participants also commented that seeing the streets empty and silent evoked feelings of loneliness and anxiety.

As for fear of going out due to concerns over infection, 30% of participants explained that they felt worried about going out, either due to fear of the disease itself, because they were at greater risk, or because they had relatives who were at risk.

*But I was more afraid to go out in case I caught it [COVID-19] than of not going out.* Woman, aged 55.

c. Outdoor exercise

More than half of the participants spoke of how their exercise routines were negatively affected by not being able to leave the house, not even to go for a walk. Some said that walking helped them to ‘clear their heads’ and feel more cheerful by distracting them from problems. The benefits of sunbathing were also mentioned, with people stating that they tried to walk and sunbathe at the window or balcony.

*For me, the thing that helped boost my mood the most was going for a walk and getting some sun at the window or on the balcony, 5 or 10 minutes, just letting the sun shine on my face, and when I was going to the shops, I’d try to walk in the sunlight*. Woman, aged 67.

Despite being locked down at home, some participants kept walking in their houses in an attempt to maintain their previous routines.

*Personally, in terms of my body, what I noticed the most was that I should have gone out walking from the beginning, because I have degenerative arthrosis. My body deteriorated, I got much more tired, and I felt angry and very annoyed. I felt the impact of not being able to go out for quite a while, I felt frustrated.* Woman, aged 56.

### Community, safety, and social networks

d. The ups and downs of living together in the same space

Ten participants stated that living with their partners and/or other relatives was a source of support and well-being. Children were also a source of support for seventeen participants.

*Now that I have my kids, I am much better. My daughters have helped me get through it. They make me see that nothing is wrong with me and that you have to try to normalise it as much as possible.* Man, divorced, aged 39.

For other participants, cohabiting during the strict lockdown was difficult since family members had to spend ‘too much time together’ with no opportunity to disconnect from each other. Living together was more problematic with certain relatives.

*The worst thing has been living with my mother, who has not lived with me for such a long time in years.* Woman, aged 36.

Ten participants lived alone. The lockdown was not a problem for some of them since they were used to it and accepted the situation. However, others reported feeling lonely and unwell as a significant part of their daily routine involved seeing relatives and friends.

*Since my husband’s passing, I’ve gone to my daughter’s on Saturdays and to my son’s on Sundays. Since this [quarantine] began, well, there’s been nothing, they don’t come here, and I don’t go there […].* Woman, aged 75.

e. Family and social support

Some participants reported that the support of their family members (although they did not live together) was critical to maintaining their mental well-being. Being supported and supporting others was perceived as positive. In addition, becoming aware of the people who are always with them and caring for them made them feel appreciative and strengthened their relationships. One woman who worked as a nursing assistant stated that she did not allow herself to be sad or cry to avoid worrying her family. This thought helped her keep her spirits up.

Two participants lamented that they could not say goodbye to their deceased loved ones, visit ill relatives and grieve with family members at funerals. They explained that the cancellation of these face-to-face events meant that people could not feel physically supported by their relatives at the most challenging of times.

*What was hardest for me was when my uncle passed away. Not being able to be there physically was very hard.* Woman, aged 39.

For people who lived alone, loneliness was exacerbated by not visiting their family or friends. Participants who live alone emphasised the importance of their social life, mainly because they tend to overthink when they are alone, and they get distracted when they are with other people. In other words, being with others improved their emotional well-being.

f. Caring as extra work

For employed women with small children, the impact of motherhood and lockdown was influenced by the number of children they had, and their age. Home-schooling placed an additional burden on parents, since they had to take care of their children 24/7. Additionally, the combination of teleworking and home-schooling turned any work-life balance upside down, and some participants described an inability to completely disconnect from work/caring responsibilities:

*One of the obstacles to following my routines that were beneficial to my mood was having a small child. I needed to pay attention to him, and I stopped doing certain things.* Woman, divorced, aged 39.

g. Digital technologies

As we can deduce from the previous paragraph, one of the most significant impacts of COVID-19 was related to socialising. Half of the participants said that not seeing or having physical closeness with some relatives was the worst aspect of lockdown.

*The hardest thing for me was not seeing my children. We saw them on video calls, but it is not the same. Now they can come over, once a week, they have lunch at home with me. We have missed that.* Man, aged 60.

For some participants, technologies served to alleviate the suffering of not physically being with their loved ones and friends by providing an online connection. They could see them and chat to them:

*I have kept my spirits up because I have not lost contact with my friends. I played games with them in a video call, or we called each other and worked on our End-of-Degree Projects together as if we were in the library*. Man, aged 22.

Nevertheless, digital technologies came with challenges. Some older adults expressed difficulties handling mobile devices, stating clumsiness and the significant differences to face-to-face contact. As far as health is concerned, some participants stated that not being able to visit their GP face-to-face was very limiting, as they believe that the quality of care decreased, and it was difficult for them to communicate what was wrong with them verbally.

*The medical appointments have been the worst thing I have dealt with. Having to be assessed over the phone… I have not handled it very well. How could I tell them that my shoulder hurts and where exactly! That is the worst situation I was confronted with.* Woman, aged 71.

Indeed, the lack of physical touch was missed too.

*Not seeing my family was the hardest thing for me. Also, my great-granddaughter was born […] So, I have suffered a lot from not seeing her. Now they have brought her to me but with a distance of a metre or two, without being able to touch her, without being able to kiss her.* Woman, aged 83.

Technologies were used as a source of information on world affairs. Some participants reported that bad news in the media about COVID-19 infections and deaths greatly influenced their state of mind, even made them cry when they saw the death toll. Some complained that the images that appeared on television made them feel bad at thinking that some of their relatives at risk could be infected. For these reasons, some avoided watching TV. Although they did not feel a direct threat from COVID-19, other participants also reported feeling bad for people who had been infected or had lost a family member. This feeling would be one of empathy for what the general population was experiencing.

*I thought continually, who remembered those who had died? I could not get it out of my head that they died alone, that their family could not see them, that at funerals they only let them stay for 10 minutes… I got really anxious and depressed… it was horrible.* Woman, aged 66.

h. Home as a privilege and leisure-time activities

A quarter of participants reported feeling well during lockdown since they were comfortable being at home. Some young and mature adults defined themselves as home-loving people, and so they did not notice much change compared to their previous life.

*During lockdown I have been very well because we work on the computer so during the week the days passed quickly. Maybe the weekends were a bit more of a drag, but I did relaxation, meditation. The bad thing was later when they said that you could go out, I no longer felt like it… I was so comfortable.* Woman, aged 62.

Some participants stated that they did not dare to go outside and defined their apartment as a refuge. Also, they said that they found it difficult to leave home pre-COVID, so the quarantine allowed them to feel more relaxed since they did not have any schedule. Some participants said that it had been good for them to have so much time to themselves and be active at home doing manual jobs. Several women stated that they felt calmer because they knew they could not go out, nobody called them [to go out], and they were fine with that. They also would have liked it to last longer, as they did the same things that they would have done if lockdown had not been in place. In terms of routine changes, fifteen participants reported having adapted very well to lockdown, adapting to their new routines. Some participants reported feeling better in lockdown, as they felt more comfortable not having any obligations:

*Lockdown has not affected me, on the contrary, it has been very good for me. I feel very well, very focused and very serene. Since I don’t like going out, and have not felt compelled to do so, I have been living a full life with my daughter and my husband. It seems contradictory to the situation we have been living in.* Woman, aged 51.

### Healthcare services

Thirty-five participants reported feeling satisfied with the performance of the healthcare system during lockdown. Some participants expressed that the health system had enough work caring for people sick with COVID-19 and regarded their own health problems as not being so serious. They did not mind waiting to be seen by a doctor and highly valued the healthcare workers’ professionalism. Some participants also spoke of budget cuts, recognising that the healthcare system could not keep pace with demand due to a lack of resources. However, at the same time, a few people suggested ways to improve healthcare, and some others reported feeling neglected:

*You call the health centre, they don’t answer, or when they answer, a machine speaks to you saying “please call later, we cannot assist you at the moment”. For the elderly, or people who are a little nervous, that machine is killing us. The social worker, I call her and she does not answer, and I need to be seen right now, so I can eat. […] I do not have money for next month, nor for this month.* Woman, aged 56.

Mental healthcare was a recurrent theme. Twelve participants suggested that psychiatric and psychological mental healthcare was lacking, especially during COVID-19, and mentioned the long waiting lists. Participants explained that many people had to resort to private care, which is expensive. They demanded more psychological help from public health services, settling for online or telephone appointments if face-to-face appointments were not possible. Feelings of empathy appeared again in people’s responses, as some of them emphasised how important mental health is, especially among people who have lost a relative during lockdown and have not been able to say goodbye in the usual way (i.e., being physically present in the company of relatives).

Twelve participants acknowledged having felt well-cared-for; some reported that they understood the extreme pressure the health system was under, which they said justified the neglect of mental health due to the circumstances of the pandemic. They demand closer monitoring but recognise that there are many people and that there were other priorities. Furthermore, they highlight the work of mental health associations, since thanks to them, they did not feel so alone and helpless.

### Hopelessness and hope

Specific depression symptoms such as pessimism could lead the individual to have a more negative view of events. Some participants have a negative view of what happened, with biases towards perceiving only the negative. They said that this situation was bad and that it would worsen, and they were getting worse and worse.

*I continue to feel anguish at all this that is happening and that has not yet ended, I am aware that we are existing without living*. Woman, aged 66.

In contrast, some participants highlight that their ability to cope, optimism, and adaptation has made them feel better and get a better perspective on the circumstances. They felt the pandemic made them realise that they must do things now rather than leaving them for tomorrow, as tomorrow is uncertain. It also helped them to pay more attention to positive things. The way people viewed their circumstances was influential in other cases, where social circumstances, and especially the perception people had of their personal situation, improved over time:

*What has made me feel better has been slowing down, because before the quarantine I didn’t feel great, I was very badly stressed from work and everything. The break has helped me and although it has been hard ... before I had no time for anything and now, I have been able to spend time with my son and spend time at home.* Woman, aged 32.

## Discussion

The results reported here highlight the role of SDHs in depression in times of COVID-19 and allow us to understand how people prioritise their needs. SDHs interact with existing non-communicable diseases (NCDs), health-related practices, and social and community networks [49]. Findings show how this interaction is exacerbated in pandemic times, as the measures adopted by the government have an immediate and unequal impact on mental health due to housing conditions, working conditions and gender-based inequalities [50].

According to participants’ perceptions of their experience of depression, the living environment is one of the most influential SDHs. It is closely linked to housing, physical space and the neighbourhood [12, 51]. This study found that people living in apartments or houses with balconies or a garden could enjoy the benefits of being “out on the street” such as walking, sunbathing, and socialising with neighbours. This corroborates existing evidence suggesting that poor housing conditions, including overcrowding and little access to outside or green space, are detrimental to physical and mental health [50].

Due to Spain’s first strict lockdown lasting seven weeks, the outdoors (*la calle*) lost its socialisation function. For many participants it also lost its therapeutic function as it was associated with the spread of the virus. Fear is one of the central emotional responses during a pandemic, and it has been fuelled by the negative news coming out in the media [39]. Yet participants found ways of bringing the qualities of the outdoors into the safety of the private sphere by creating new spaces for socialising such as balconies.

When queried about health-related practices, most of the participants were concerned about their lack of routine. Some greatly missed their physical activities such as walking. [52, 53]. Those who could walk on their balconies believed their mental health improved due to more exercise [17, 54]. Walking and synthesising vitamin D thanks to sun exposure have antidepressant effects both as a consequence of biochemical processes and cultural understandings of the outdoors [55]. Additionally, regular exercise prevents heart disease and, by limiting obesity, reduces the onset of diabetes, promotes a feeling of well-being, and protects the elderly from depression [24].

Future management of epidemics should take into account the importance of the living environment. Policies and public and private spaces should be provided to enhance social connectedness and enable people to access blue and green spaces, as these are associated with improved mental health in adults. Physical activity in green space is more beneficial than activity in other settings [56]. Findings confirm existing evidence that low-income areas are hit harder by the epidemic as they tend to have a lack of space [50] – in this case, balconies or gardens that integrate the benefits of the outdoors (nature and people) into the safety of the home.

The next SDH that emerges from the results is community, safety, and social networks. Social and community networks significantly impact our sense of life satisfaction and well-being [57]. Friendship, good social relationships, and strong support networks improve health at home, at work, and in the community [24].

Living together with family was described as an advantage for the majority of participants. These results align with Günther-Bel et al.’s study [58], which found that family dynamics during quarantine had improved rather than deteriorated. However, relationships appeared more harmonious when there were no children in the household [58]. Our data also shows that families with teenagers faced some tensions. What this means is that cohabitation is a double-edged sword. On the one hand, caring for others in the home can be beneficial because it helps to be active and have company. On the other hand, it can be experienced as a burden, negatively affecting both our health and the quality of care provided [59]. This may be due to the combination of emotional and physical fatigue that caring entails, increased by the pandemic situation [60], lack of resources and space and the suddenly enforced proximity with immediate family [39], especially if relationships were already strained. The weight of social norms and gender inequalities means that female caregivers, particularly low income or single parents, still experience greater levels of burden [61, 62]. We must highlight that social support protected against this burden and led to greater satisfaction with care [61].

This recommendation is even more relevant if we take into account that women are more likely to be diagnosed with depression. In addition, women suffer more from symptoms derived from quarantine measures than men [27, 63, 64]. This gender gap is widely reported in adults, with working life and family roles having a greater impact on women’s mental health [65].

For people in one-person households, loneliness was accentuated, with a consequential decline in mental well-being [66]. Loneliness (perceived as social isolation) negatively affects physical and mental health [39, 57]. Self-isolation policies can increase social isolation and relationship difficulties [39]. Social isolation might be prevented by increasing the amount of contact with peers, or by sharing a common interest with others, as this could give people a sense of belonging to a community [67].

With regard to the use of digital technologies, quality (e.g. feeling ‘close’) was more strongly valued than quantity (e.g. dehumanised-mechanic video calls with professionals), and those with higher quality or more face-to-face or phone/video contact had fewer depressive symptoms [68]. There are also differences among seniors’ perspectives and preferences when using technologies [69], making the digital literacy gap more evident [70]. Technology was important for the older population during the pandemic, facilitating meaningful relations [71]*.* However, the use of technologies does not fully replace traditional ways of socialising that involve ‘closeness’ and even physical touch or experiences such as kissing, hugging, or face-to-face conversation, which are valued positively in Western Europe [39].

When looking at the home as a privilege, and leisure activities, people who were usually more sociable or had higher empathy had more depressive symptoms during enforced reduced contact [68]. Yet, for people with depression socialising can be challenging (e.g. due to experiencing feelings of exclusion) [72]. Besides that, some participants have changed their view of life, paying more attention to other aspects they had neglected, such as spending more time with their families or taking up leisure activities. This means that they consider being at home as a privilege, instead of all the negative feelings they might also feel [20]. Stressful times such as the ones we are living through could help us to reorganise our priorities, and lead to deeper relationships and a greater appreciation of life [73]. Therefore, positive emotions also emerged due to increased leisure time and the slower pace of life during lockdown [74].

Access to healthcare is another SDH that emerged in this study. Healthcare systems take on the role of identifying and addressing patients’ unmet social needs, making inroads into improving population health and health equity [75]. Examples of health system interventions include additional care and support for disadvantaged patients, additional resources for rehabilitation programmes to reduce the effects of illness on people’s earning potential, and equitable healthcare financing [12]. Previous physical and mental health conditions have increased inequalities because of reduced access to healthcare services for non-COVID-19 reasons [50]. Most of our participants felt satisfied with the health system's performance during lockdown, although several mentioned the lack of mental healthcare. The general satisfaction with healthcare services might be because of health workers’ image in the media as “heroes” who deserve all the respect in the world for the great effort and work they are undertaking during the pandemic. Conformity with restrictions and the understanding shown by the population may be due to the appeal for sacrifice and cooperation by governments and the media for the good of society in general [39]. As in other studies, a strong sense of communal or civil responsibility was found [76]. Nonetheless, there is a demand for more care from the public health system; since if people have received care, it has been mainly through private associations with state-funded financial support. Mental healthcare is critical as it impacts the rest of the family. The family covers the care systems’ deficiencies and weaknesses, leading to overburdening and diminishing quality of life for caregivers [77].

### Recommendations, implications for policy and practice

There is a need to develop specific strategies to address or mitigate SDHs to reduce health inequalities [78]. For example, our study’s results might have implications for urban planning, as we have seen the importance of adequate home size and some exposure to open air (i.e., balconies or terraces).

Governments should support families with young children, caregivers or people with physical or mental health problems, as people with depression are one of the groups that place the most significant burden on their caregivers [79]. Furthermore, community activities to combat social isolation should be promoted [32], as in possible future times of distress and crisis, human resilience depends on the richness and strength of social connections and active engagement in groups and communities [57].

In our study we focus specifically on people with depression. This field of research is essential as it has been acknowledged that people with mental illness and their families should participate in developing policies and thus contribute to strengthening mental healthcare systems worldwide [77]. Recommendations related to mental health would be a shared approach to vulnerability between public health services, primary care centres, hospital services, municipal health services, occupational health and mental health facilities [32]. Professional services are also needed to support people across the psychological disciplines, with face-to-face and online access and more accessible referral routes into these services as well as better connectivity with GP practices [17], especially for those with chronic diseases, diagnoses of mental health disorders, disordered substance use and highly complex patients [32]. Another recommendation regarding the management of negative emotions during quarantine periods would be providing people with clear information and basic necessities, as well as appealing to the common good [20].

### Strengths and limitations

Despite the specific characteristics of our sample (people living with depression), the results could be highly relevant to the general population for two reasons. Firstly, our holistic approach based on the SDH framework explored people’s experiences of lockdown as a whole, without assuming that people with depression would be more affected than people without depression. Secondly, our approach did not assume that some SDHs are more important than others and the analysis path was inductive. However, interviews were done by telephone due to quarantine constraints, and some non-verbal information may have been lost. Another limitation would be that inherent to the type of study, which cannot be generalized to the entire population with mental disorders.

## Conclusions

This study provides further evidence about how SDHs have played a fundamental role in shaping the experiences of people living with depression during COVID. Housing and working conditions, physical and mental health, and access to health services, health-related practices, and social and community networks were recurrent themes. The impact that these SDHs have on our mental health should not be disregarded, and governments should consider implementing social welfare programmes to tackle both psychosocial problems and material needs.

## Data Availability

The datasets generated during and/or analysed during the current study are not publicly available due to the personal statements that have been said by the participants but are available from the corresponding author on reasonable request.
